# Associations between health-related quality of life, physical function and pain in older women with osteoporosis and vertebral fracture

**DOI:** 10.1186/s12877-019-1268-y

**Published:** 2019-11-04

**Authors:** Brita Stanghelle, Hege Bentzen, Lora Giangregorio, Are Hugo Pripp, Astrid Bergland

**Affiliations:** 1 Institute of Physiotherapy, OsloMet - Oslo Metropolitan University, PO Box 4, St. Olavs Plass, 0130 Oslo, Norway; 2 Leader of the Institute of Physiotherapy, OsloMet - Oslo Metropolitan University, Oslo, Norway; 3 Department of Kinesiology, University of Waterloo and Schlegel-UW Research Institute for Aging, Waterloo, Canada; 4 Faculty of Health Sciences, OsloMet - Oslo Metropolitan University, Oslo, Norway; 5 Institute of Physiotherapy, Leader of the Research Group Age, health and Welfare, Oslomet - Oslo Metropolitan University, Oslo, Norway

**Keywords:** Osteoporosis, Vertebral fracture, Health-related quality of life, Physical function, Pain

## Abstract

**Background:**

Osteoporosis and vertebral fractures represent a major health burden worldwide, and the prevalence of osteoporosis is expected to increase as the world’s population ages. Suffering from vertebral fracture has a substantial impact on the individual’s health-related quality of life (HRQoL), physical function and pain. Complex health challenges experienced by older people with osteoporosis and vertebral fractures call for identification of factors that may influence HRQoL, as some of these factors may be modifiable. The objective is to examine the independent associations between HRQoL, physical function and pain in older women with osteoporosis and vertebral fracture.

**Methods:**

This study has a cross-sectional design, using data from 149 home-dwelling Norwegian women with osteoporosis and vertebral fracture, aged 65+. Data on HRQoL (Short Form 36 (SF-36), Quality of Life Questionnaire of the European Foundation for Osteoporosis (QUALEFFO-41)), physical function (walking speed, balance and strength), pain, as well as sociodemographic information were collected. Simple linear regression analyses were conducted and multivariable regression models were fitted to investigate the associations.

**Results:**

Lower levels of HRQoL were significantly associated with lower levels of physical function, measured by walking speed, and higher levels of pain. Pain was significantly associated with all of the subscales in SF-36, with the exception of Mental Health and Mental Component Score, and all the subscales of QUALEFFO-41. Walking speed was significantly associated with 5 of 8 subscales of SF-36 (except Bodily Pain, Vitality, Mental Health and Mental Component Score), and with 4 of 6 subscales of QUALEFFO-41 (except Score Pain and Mood).

**Conclusion:**

This study shows that pain and walking speed were, independently of one another, associated with HRQoL in older women with osteoporosis and vertebral fracture. These findings can inform clinicians and health managers about the importance of pain management and exercise interventions in health care for this group. Future research should address interventions targeting both physical function and pain with HRQoL as an outcome.

**Registration:**

ClincialTrials.gov Identifier: NCT02781974. Registered 18.05.16. Retrospectively registered.

## Introduction

Osteoporosis is a major health burden worldwide [1], affecting more than 22 million women and 5.5 million men in the European Union [2]. Osteoporosis is common among older populations and affects people of both genders, but is more prevalent in women [1, 3, 4].

The disease is associated with an increased risk of fracture, and vertebral fractures are among the most common type of osteoporotic fracture [1, 5]. Vertebral fractures may cause severe pain and loss of function, but can also present with mild or no symptoms [5]; this may explain why only 30% of these fractures come to clinical attention. Fractures of this nature have a substantial impact on the individual’s health-related quality of life (HRQoL) [6].

HRQoL is a multidimensional concept that encompasses the physical, psychological, social and somatic domains of functioning and well-being [7, 8]. In addition, HRQoL may offer prognostic benefits for prediction of clinical complications and mortality [9]. The need to improve HRQoL in older people is widely acknowledged [10], and identification of variables associated with HRQoL is a prerequisite for such effort [11]. It is well documented through research that people with osteoporosis who have suffered vertebral fracture experience poorer HRQoL compared to those who do not have osteoporosis or have not experienced vertebral fracture [6, 8, 12, 13]. Vertebral fractures are particularly associated with reduced HRQoL in its physical domain, as well as with pain and reduced physical function [13].

Kerr et al. [14] state that osteoporosis and fracture can have a profound impact on physical function, and that this impact accumulates over time. The experience of fracture may lead to a downward spiral of decline in physical function due to pain and loss of bone and muscle strength. This may in turn result in decreased mobility, activity restriction and reduced ability to carry out daily activities, and increased risk of new fractures.

Among the older population generally, impaired function is a predictor of reduced HRQoL [15]. In elderly men and women with osteoporotic fractures, lower quality of life was associated with reduced walking speed [16]. As far as we know, only one study has investigated the relationship between HRQoL and physical function in people with vertebral fracture in particular. A study by Bergland et al. [17] (*n* = 89) on older women with osteoporosis and vertebral fracture found that those in the 75% group with the highest maximum walking speed and those in the 75% group with best performance on balance had better scores on HRQoL compared to those in the 25% group with lowest maximum walking speed and poorer performance on balance [17].

Pain is another factor that may influence HRQoL [18]. Back pain is common in osteoporosis patients, even where there is no history of vertebral fracture, and research has revealed a negative association between back pain and balance, as well as mobility [19]. Furthermore, pain intensity is found to have a negative impact on physical HRQoL, walking speed, balance and leg strength in women with osteoporosis with and without vertebral fracture [20].

In summary, people with osteoporosis and vertebral fracture experience reduced HRQoL, physical function and increased pain. HRQoL is a key indicator of people’s health status, and identification of variables associated with it is pivotal in preventing decline in these individuals’ HRQoL. To our knowledge, this study is the first to study the relationship between HRQoL, physical function and pain in a population of older women with osteoporosis and vertebral fracture. This knowledge is key, since we know that some of these factors may be modifiable. Identification of factors that account for variations in HRQoL among people with vertebral fracture may guide intervention strategies for enhanced HRQoL in this patient group. Decline in physical function and the associated lower HRQoL are reported to respond positively to exercise interventions [21, 22]. Additionally, a recent review concluded that pain after osteoporotic fracture requires a multifaceted approach, including both pharmacological (i.e., pain medication) and non-pharmacological (e.g. physical exercise, physiotherapy) interventions [23].

This study’s objective is to examine the independent associations between various HRQoL subscales and physical function (i.e., walking speed, balance, muscle strength) as well as pain and sociodemographic factors in older women diagnosed with osteoporosis and vertebral fracture. To perform a comprehensive investigation we applied both a generic and a disease-specific HRQoL instruments to examine differences between the instruments’ associations with physical function, pain and sociodemographic factors [6, 7]. We hypothesized that those with poor generic and disease-specific HRQoL had lower levels of physical function and higher levels of pain.

## Methods

### Study design

The study uses cross-sectional data from the baseline measurements of a randomised controlled trial conducted between 2016 and 2018 [24]. The trial was registered at ClinicalTrials.gov in May 2016 (registration number NCT02781974). The recruitment period was from January 2016 to April 2018. The STROBE guidelines are followed in our report on the design, analysis and presentation of data [25].

### Setting and participants

The present study was undertaken at Oslo Metropolitan University (OsloMet) and at a sports and physiotherapy clinic in the Oslo area. Participants were recruited from three different outpatient clinics in and around the city of Oslo, Norway. The recruitment plan has been described elsewhere [24]. Data from 149 women are included in the final sample that was analysed in this study. We included women aged 65 years or older, who were living at home and able to walk independently with or without a walking aid. Further, to be found eligible, the women had to be diagnosed with osteoporosis and have a T-score of − 2.5 standard deviations (SD) or under at the femoral neck, lumbar spine or both [26], verified by Dual X-ray Absorptiometry (DXA) scan. In addition, they had to have at least one previous vertebral fracture classified grade 1, 2 or 3 [27], verified on DXA or X-ray by a trained medical doctor. Exclusion criteria included inability to speak and understand Norwegian, self-reported severe diseases or other health conditions that made it unsafe to exercise, such as severe chronic obstructive pulmonary disease or a progressive neurological disease. In cases of uncertainty, the women were asked to consult their physician to verify that it was safe for them to exercise.

### Outcome measures

#### Sociodemographic and background variables

A self-reporting questionnaire was completed by all participants, and included questions regarding age, education (years at school), whether they lived alone (yes/no), body mass index (BMI) and comorbidity (defined as four or more self-reported diagnoses).

#### HRQoL

In this study, HRQoL was measured by both a generic and a disease-specific self-reported questionnaire. Generic HRQoL instruments are designed to be applicable across different populations and conditions, but may not always be as sensitive to the subtle effects or variations of a specific condition as a disease-specific instrument may be [7]. The generic questionnaire Short Form 36 (SF-36) is widely used across various populations for diagnosis [28], and has been validated and translated into Norwegian [29]. The SF-36 is divided into eight subscales: physical functioning (PF), role limitations-physical (RP), bodily pain (BP), general health (GH), vitality (VT), social function (SF), role limitations-emotional (RE) and mental health (MH). The instrument has no overall total score, but a physical component score (PCS) and mental component score (MCS) are calculated. The subscales are scored from 0 to 100: the higher the score, the better the health status [30]. The SF-36 has been shown to have high reliability and validity for the assessment of older people [31].

The frequently used Quality of Life Questionnaire of the European Foundation for Osteoporosis (QUALEFFO-41) is a disease-specific questionnaire that was developed to assess people with osteoporosis and vertebral fracture [6]. Disease-specific HRQoL instruments are considered more valid in the sense that they may measure HRQoL more accurately in that particular disease [6]. The instrument contains 41 questions or items in five subscales; Score Pain, Physical Function, Score Leisure and Social Activities, Views about Health in General and Mood. These five subscales may be evaluated separately or represented within a total score of all 41 items [6], the QUALEFFO total score. The scores from the various domains are calculated on a scale of 0–100, where 0 represents the best and 100 the worst quality of life.

#### Physical function

The physical function tests were assessed by experienced physiotherapists who all went through a training program regarding testing procedures to ensure consistency in performing the tests. The multidimensional concept of physical function is here defined as an individual’s capacity to carry out the physical activities associated with daily life, reflecting dimensions that include motor control, physical fitness and habitual physical activity [32]. The physical function measurements represent balance, walking speed and upper- and lower-limb muscle strength. Functional Reach (FR) is a measure of balance [33], and is strongly connected to physical frailty [34]. It is a test on capacity to reach forward in an anticipatory postural adjustment task [35]. It measures in centimetres the maximal distance one can reach forward from a static standing position. Walking speed was assessed by a 10-m walk, whereby the women were instructed to walk that distance at their habitual walking speed [36]. Muscle strength in the upper limb was assessed by measuring the number of arm curls performed over 30 s holding a manual of 5 pounds (2.27 kg) [37]. For the lower limb, the 30-s sit-to-stand (STS) test was applied, counting the number of times the participant could go from a seated to a standing position over 30 s [37].

#### Pain

Pain is a multidimensional concept, and the International Association for the Study of Pain (IASP) defines pain as “an unpleasant sensory or emotional experience associated with actual or potential tissue damage, or described in terms of such damage” [38]. Global pain intensity was measured according to the Numerical Rating Scale (NRS), indicating the participants’ pain levels within the preceding 7 days (0 = no pain, 10 = unbearable) [39].

#### Analysis

Statistical analyses were conducted using SPSS version 24. Continuous variables were described with mean, standard deviation (SD), minimum and maximum, and 95% confidence interval. Categorical variables were described with percentages. Skewness was examined using histograms, boxplots and comparison of the mean and the median values. A floor or ceiling effect was considered when more than 20% had the lowest or highest possible score [40]. Initially, univariable linear regression was used to explore the associations between the different subscales of SF-36 and QUALEFFO-41 as the dependent variable, with each of the sociodemographic background variables and the variables of physical function and pain as the independent variables. Furthermore, using a data-driven and explorative statistical approach, a set of multivariable backward regression models were fitted for the different subscales using backward variable selection to examine the strength of their association with the various variables of sociodemographic background, physical function and pain. Multicollinearity was inspected by correlation of the independent variables prior to multivariable regression analysis, and no presence of multicollinearity was found [41]. Variables for the multivariable regression models were selected using backward variable selection with a *p*-value ≤0.20 as criteria for elimination, as recommended for better prediction and greater power for the selection of prediction variables [42]. The unstandardized and standardized regression coefficients, *p*-values and the model’s fit estimated adjusted R^2^ were reported. Residuals were examined, and the criteria for multivariable regression were met.

## Results

The participants’ mean age was 74.2 years (SD 5.8) (Table 1). The mean number of years of school attendance (education) was 13.1 (SD 3.4). Of the participants, 45.1% were living alone, and the mean score for pain (NRS) in the preceding week was 3.4 (SD2.5). Mean BMI was 23.2 kg/m^2^ and the presence of comorbidities, defined as 4 or more self-reported diagnoses, was found in 40.5% of the participants. The mean walking speed was 1.21 m/s (SD 0.30). Regarding the HRQoL instruments, the subscale with the best score in SF-36 was SF (mean 84.1, SD 20.5) while in QUALEFFO-41 the highest score was observed in Physical Function (mean 17.2, SD 13.2). In SF-36’s subscale SF, a ceiling effect was found in the 52% who achieved the top score of 100.

**Table 1:** Characteristics of the study population. Means, standard deviations, percentages, minimum - maximum and 95% confidence interval

Sociodemographic and descriptive characteristics	Mean (SD)	Min-max	95% CI
Age, mean (SD)	74.2 (5.8)	65–89	73.3–75.1
Education, in years, mean (SD)	13.1 (3.4)	7–22	12.6–13.7
Living alone Yes %	45.1		
No %	54.9		
BMI, mean (SD)	23.2 (3.7)	15.8–35.3	22.6–23.8
Comorbidity Yes %	40.5		
No %	59.5		
Pain last week, NRS, (0–10), mean (SD)	3.4 (2.5)	0–10	2.9–3.8
Pain categories, % mild (0–3)	52.1		
% moderate (4–6)	36.1		
% severe (7–10)	11.8		
Physical function			
FR cm, mean (SD)	34.1 (6.4)	18–56	33.1–35.1
Walking speed m/s, mean (SD)	1.21 (0.30)	0.30–1.94	1.16–1.26
Armcurls, mean (SD)	15.2 (3.8)	4–28	14.5–15.8
30sSTS, mean (SD)	12.6 (3.9)	1–26	12.0–13.3
HRQoL			
SF-36, mean (SD)^a^			
Physical functioning (PF)	67.6 (22.9)	10–100	63.8–71.3
Role physical (RP)	63.0 (29.0)	0–100	58.3–67.7
Bodily pain (BP)	58.8 (23.7)	10–100	54.9–62.6
General health (GH)	63.7 (23.3)	10–100	59.9–67.5
Vitality (VT)	53.9 (16.6)	10–90	51.2–56.6
Social functioning (SF)	84.1 (20.5)	25–100	80.7–87.4
Role emotional (RE)	63.1 (20.6)	0–80	59.8–66.5
Mental health (MH)	71.6 (13.2)	24–88	69.5–73.8
Physical Component Score (PCS)	43.0 (10.0)	18.8–62.7	41.4–44.6
Mental Component Score (MCS)	49.7 (6.6)	24.8–62.0	48.6–50.8
QUALEFFO-41, mean (SD)#			
Score Pain	35.3 (25.2)	0–95	31.2–39.4
Physical Function	17.2 (13.2)	0–55.9	15.1–19.4
Score Leisure and Social Activities	25.8 (21.1)	0–95	22.4–29.3
Score Views about Health in General	44.8 (22.5)	0–100	41.0–48.6
Score Mood	34.3 (12.9)	8.3–75	32.2–36.4
Total Score Qualeffo	26.7 (13.1)	7.5–65.3	24.6–28.8

*SD* Standard deviation, *CI* Confidence Interval, *BMI* Body Mass Index, *NRS* Numerical Rating Scale, *FR* Functional Reach; 30sSTS 30 s Sit to Stand; ^a^Score 0–100, 100 best score; #Score 0–100, 0 best score

Additional file 1 shows the results of the univariable linear regression analyses. The univariable analyses show that pain was significantly associated with all subscales of both SF-36 (with the exception of MCS) and QUALEFFO-41 (standardized β ranged from − 0.77 to − 0.24 in SF-36, and from 0.76 to 0.28 in QUALEFFO-41). Walking speed was also significantly associated with all subscales of both SF-36 (with the exception of MCS) and QUALEFFO-41 (standardized β ranged from 0.67 to 0.22 in SF-36, and from − 0.62 to – 0.24 in QUALEFFO-41). The highest values of the standardized βs were observed in the associations between the independent variable pain and the BP subscale in SF-36 and the Score Pain subscale in QUALEFFO-41 (Additional file 1).

Tables 2 and 3 present the results from the multivariable linear regression analyses after backward variable selection. The variables that were assessed as associated with SF-36 and QUALEFFO-41 (*p* ≤ 0.20) are presented for each subscale of the two HRQoL instruments. Pain was significantly associated with all subscales of SF-36 (Table 2) and QUALEFFO-41 (Table 3), with the exception of MH and MCS in SF-36. Pain had the highest standardized β in BP in SF-36 and in Score Pain in QUALEFFO-41, as expected. The standardized β was - 0.70 for BP and 0.70 for Score Pain. Walking speed was significantly associated with several of the subscales of SF-36 (Table 2) and QUALEFFO-41 (Table 3), with the exception of BP, VT, MH and MCS in SF-36 and the QUALEFFO-41 Score Pain and Mood subscales.

**Table 2:** Associations between SF-36, physical function and pain (multivariable regression)

		Standardized β	*P*-value	B (95% CI)	Adjusted R^2^
Physical Functioning	Pain	−0.29	**< 0.001**	−2.83 (− 3.95 to – 1.71)	0.596
	Functional reach	0.21	**0.001**	0.78 (0.31–1.25)	
	10 m walking speed	0.41	**< 0.001**	32.48 (20.67–44.28)	
	30 s Sit to Stand	0.14	**0.047**	0.83 (0.01–1.66)	
Role Physical	Pain	−0.45	**< 0.001**	−5.36 (− 6.88 to − 3.84)	0.502
	Comorbidity	0.14	**0.029**	8.34 (0.85–15.82)	
	Functional reach	0.16	**0.023**	0.75 (0.11–1.40)	
	10 m walking speed	0.29	**< 0.001**	28.85 (14.07–46.63)	
Bodily Pain	Pain	−0.70	**< 0.001**	−6.71 (−7.78 to – 5.64)	0.615
	Comorbidity	0.14	**0.030**	6.47 (1.19–11.75)	
	Functional Reach	0.08	0.198	0.30 (−0.16–0.76)	
	10 m walking speed	0.10	0.148	7.68 (−2.75–18.10)	
General Health	Pain	−0.26	**0.002**	−2.52 (−4.07 to − 0.98)	0.170
	10 m walking speed	0.26	**0.002**	21.02 (8.18–33.86)	
Vitality	Pain	−0.31	**< 0.001**	−2.14 (−3.20 to – 1.07)	0.246
	Comorbidity	0.21	**0.008**	7.19 (1.91–12.47)	
	Functional reach	0.14	0.109	0.37 (− 0.08–0.83)	
	10 m walking speed	0.12	0.183	7.07 (−3.38–17.53)	
Social Functioning	Living condition	0.15	0.063	5.04 (−0.33–12.20)	0.209
	Pain	−0.25	**0.003**	−2.08 (−3.42 to − 0.74)	
	Comorbidity	0.19	**0.017**	7.98 (1.45–14.50)	
	10 m walking speed	0.19	**0.022**	12.74 (1.85–23.63)	
Role Emotional	Education, years	0.20	**0.014**	1.17 (0.24–2.10)	0.229
	Pain	−0.25	**0.003**	−2.06 (−3.41 to – 0.71)	
	Comorbidity	0.12	0.151	4.84(−1.79–11.47)	
	10 m walking speed	0.24	**0.004**	16.79 (5.33–28.26)	
Mental Health	Living condition	0.17	**0.041**	4.47 (0.18–8.76)	0.124
	Pain	−0.16	0.060	−0.85(−1.74–0.04)	
	Comorbidity	0.21	**0.015**	5.55 (1.08–10.02)	
	10 m walking speed	0.15	0.062	0.53(−0.03–1.09)	
PCS	Pain	−0.50	**< 0.001**	−2.07(−2.53 to −1.62)	0.631
	Comorbidity	0.11	**0.046**	2.27 (0.04–4.49)	
	Functional reach	0.18	**0.005**	0.28 (0.09–0.47)	
	10 m walking speed	0.26	**0.001**	8.80 (3.90–13.70)	
	30 s Sit to Stand	0.11	0.099	0.28(−0.05–0.61)	
MCS	Comorbidity	0.22	**0.008**	2.95 (0.78–5.13)	0.082
	Living condition	0.23	**0.005**	3.08 (0.94–5.23)	

*PCS* Physical Component Score, *MCS* Mental Component Score

Variables in bold were selected for the multivariable regression models using stepwise backward regression with *p*-value ≤0.20 as criteria for elimination

**Table 3:** Associations between Qualeffo-41, physical function and pain (multivariable regression)

		Standardized β	*P*-value	B (95% CI)	Adjusted R^2^
Score Pain	Pain	0.70	**< 0.001**	7.09 (5.98–8.19)	0.604
	BMI	0.10	0.076	0.66 (−0.07–1.40)	
	Comorbidity	−0.18	**0.001**	−9.08 (−14.56–3.59)	
Physical Function	Education, years	−0.13	**0.024**	−0.49 (− 0.91 to – 0.07)	0.632
	Pain	0.36	**< 0.001**	1.97 (1.36–2.57)	
	BMI	0.11	**0.042**	0.39 (0.01–0.77)	
	Comorbidity	−0.14	**0.016**	−3.66 (−6.63 to – 0.68)	
	Functional reach	−0.25	**< 0.001**	−0.52 (− 0.78 to – 0.26)	
	10 m walking speed	−0.34	**< 0.001**	−15.44 (−21.41 to-9.48)	
Score Leisure and	Education, years	−0.17	**0.006**	−1.09 (− 1.86 to – 0.31)	0.549
Social Activities	Pain	0.28	**< 0.001**	2.41 (1.31–3.51)	
	Functional reach	−0.18	**0.002**	−0.74 (−1.21 to– 0.27)	
	10 m walking speed	−0.34	**< 0.001**	−25.73 (−37.34 to-14.12)	
	30 s Sit to Stand	− 0.12	0.099	− 0.70 (−1.54–0.14)	
Score views about	Education, years	− 0.12	0.131	− 0.79 (− 1.82–0.24)	0.277
Health in General	Pain	0.31	**< 0.001**	2.85 (1.39–4.31)	
	Comorbidity	−0.15	0.054	−7.18 (−14.47–0.12)	
	10 m walking speed	−0.26	**0.003**	−19.82 (−32.55 to −7.08)	
Score Mood	Living condition	−0.16	0.055	−4.21 (−8.50–0.09)	0.104
	Pain	0.22	**0.011**	1.14 (0.26–2.04)	
	10 m walking speed	−0.14	0.107	−5.97 (−13.27–1.32)	
Total Score	Education, years	−0.15	**0.006**	−0.57 (− 0.97 to − 0.16)	0.658
	Pain	0.49	**< 0.001**	2.65 (2.07–3.22)	
	Comorbidity	−0.15	**0.008**	−3.90 (−6.74 to – 1.13)	
	Functional reach	−0.18	**0.003**	−0.39 (− 0.64 to – 0.13)	
	10 m walking speed	−0.31	**< 0.001**	−14.12 (−19.77 to – 8.48)	

Variables in bold were selected for the multivariable regression models using stepwise backward regression with *p*-value ≤0.20 as criteria for elimination

Among the multivariable models (Tables 2 and 3), the model with total QUALEFFO-41 score as its dependent variable, and living condition, pain, comorbidity, FR and walking speed as significant independent variables, accounted for 65.8% of the variance. This was the highest explained variance across all the models (Table 3). Pain was the largest unique contributor (standardized β 0.49, *p* < 0.001), while walking speed was the second largest (standardized β − 0.31, *p* < 0.001).

## Discussion

This study’s key findings show that pain and physical function were, independently of one another, associated with both SF-36 and QUALEFFO-41. These findings may have implications for the tailoring of health care interventions aimed at addressing HRQoL in older women with osteoporosis and vertebral fracture. These results support previous research that suggests that pain management and exercise interventions are important for this group [21–23].

One key finding of the present study is the significant association between HRQoL and pain, which was observed in most subscales in both SF-36 and QUALEFFO-41 (Tables 2 and 3), suggesting that pain may influence several dimensions of HRQoL. Pain after vertebral fracture is common [5], and an understanding of the complex underlying mechanisms of osteoporotic pain is key for its proper management [23]. The existing literature verifies that pain has negative influence on physical function with respect to walking speed, balance and mobility [19, 20] in women with osteoporosis. This corresponds well with the results of the present study, which found that pain was independently associated with the PF subscale in SF-36 and Physical Function in QUALEFFO-41. In addition, our findings highlight the importance of pain management. This finding is consistent with Liu-Ambrose et al. [19], who demonstrated that the high prevalence of back pain among older women with osteoporosis underscores the importance of pain management in the treatment of osteoporosis. Furthermore, regarding exercise recommendations for individuals with osteoporosis or osteoporotic vertebral fracture [43], there is limited evidence that pain is reduced after short-term (i.e., 10-week) intervention. Therefore, based on our findings, we recommend that proper pharmacological pain management be incorporated into interventions for patients with osteoporosis and vertebral fracture [23].

Another key finding is the significant association between physical function, measured by walking speed, and HRQoL. The existing literature shows that mobility is a predictor of quality of life in both older people in general [15] and in people with osteoporosis [8]. Both studies [8, 15] mentioned included men and women, and it is uncertain whether the results can be generalized to women with osteoporosis and vertebral fracture. Walking speed is recommended as a useful clinical indicator of well-being [44, 45]. In general, older people with the ability to walk faster than 1.0 m/s are considered to have good functional status, lower risk of health events, and better survival prognosis [45, 46]. The mean walking speed observed in the present study is 1.21 m/s, which may indicate that the women included in our study have relatively good physical function. The present study and the existing literature suggest that walking speed may be an important measure to consider for the maintenance and enhancement of HRQoL. Furthermore, studies have shown that exercise can improve walking speed in older women with osteoporosis and vertebral fracture [21].

The present study extends the results of a previous study on the association between HRQoL and physical function in older women with osteoporosis and vertebral fracture [17], which also observed significant associations between HRQoL and walking speed. However, measurements of pain were not included. Additionally, the sample size was smaller, and maximum walking speed was used as a measure of mobility. Thus, comparison with the present study should be made with caution. An study observed a significant association between PCS (SF-12, a shorter version of SF-36) and walking speed as well as mobility in a population of people with osteoporotic fractures [16]. Findings showed that walking speed and mobility, measured by TUG, were related to PCS of SF-12 (a shorter version of SF-36). However, the population included both men and women and the participants had suffered different types of osteoporotic fractures, not only vertebral fractures.

Furthermore, our study highlights that the strength of the association between pain and walking speed varies across the different subscales of the two HRQoL instruments. Subscales representing physical function, physical role or participation have stronger associations than do subscales representing emotional or mental aspects. This pattern can be found in both the SF-36 and QUALEFFO-41 (Tables 2 and 3). Similar results are found in studies investigating HRQoL [13, 20]. Interestingly, there were comparable results across the generic (SF-36) and disease-specific (Qualeffo-41) HRQoL instrument regarding associations with physical function and pain. The disease-specific instruments are reckoned to be more sensitive to the specific disease [6], which may indicate that the burden of disease for our study population were moderate. This is also supported by the relatively high functioning of the women. On the other hand, our study’s SF-36 scores are slightly lower than the scores of women from comparable age groups who participated in a recent study by Jacobsen et al. [47], as part of a sample representing the general population of Norwegians across age groups ranging from 18 to 90 years. This is in line with several studies reporting that living with osteoporosis and vertebral fracture affects HRQoL negatively [12, 13, 48]. Furthermore, our participants have better mean scores of QUALEFFO-41 subscales compared to the mean scores of QUALEFFO-41 subscales reported by Bergland et al. [17]. This indicates better HRQoL for our population, since lower QUALEFFO-41 scores represent better HRQoL.

This study has several limitations. First, the women included in this cross-sectional study were recruited for a randomized controlled trial aimed at potentially improving their physical function and HRQoL through an exercise programme. The participants may be fitter and a have higher level of physical function than the general population of older women who have osteoporosis and have experienced vertebral fracture. Second, all participants were living in urban areas and no men were included. This limits the generalizability of the results. Third, we have no data regarding about how many fractures the participants had, which would have facilitated the analysis of subgroups depending on the number of fractures experienced. Finally, the study is cross-sectional, and no causal relations can be established.

## Conclusion

In conclusion, this study has verified that pain and physical function are significantly associated with HRQOL, as measured using both a disease-specific and a generic HRQOL instrument, in older women with osteoporosis and vertebral fracture. This study’s findings can inform clinicians and health managers about the importance of pain management and exercise interventions in the development and organization of clinical services in health care. Future research should address interventions that can target both physical function and pain management for older women with osteoporosis and vertebral fracture.

## Supplementary information

**Additional file 1: Table S1a.** Associations between SF-36, physical function, pain and background variables. (Univariable linear regression with different subscales of SF-36 as dependent variables). **Table S1b.** Associations between Qualeffo-41, physical function, pain and background variables. (Univariable linear regression with different subscales of QUALEFFO-41 as dependent variables).

## Data Availability

The datasets generated and analyzed during the study are only available to the participating researchers due to data protection laws. Subsets or aggregation of these data will not include information that could compromise research participants’ privacy, and an anonymized subset is planned to be made available in a public repository after the project is finished. An anonymized dataset analyzed in this study can be made available from the corresponding author on reasonable request.

## Abbreviations

BMI: Body mass index
BP: Bodily Pain
DXA: Dual X-ray Absorptiometry
FR: Functional Reach
GH: General Health
HRQoL: Health Related Quality of Life
MCS: Mental Component Summary
MH: Mental Health
NRS: Numerical Rating Scale
PCS: Physical Component Summary
PF: Physical Functioning
QUALEFFO – 41: Quality of Life Questionnaire of the European Foundation for Osteoporosis
RE: Role Emotional
RP: Role Physical
SD: Standard deviations
SF: Social Functioning
SF-36: Short Form 36 Health Survey
STS: 30 s Sit to Stand
TUG: Timed Up and G
VT: Vitality
