# Supplementary material for: Associations between health-related quality of life, physical function and pain in older women with osteoporosis and vertebral fracture
Source: BMC Geriatr. 2019 Nov 4;19:298. doi: 10.1186/s12877-019-1268-y (PMC6829800; doi:10.1186/s12877-019-1268-y)
Supplement: Supplementary file 1 — Additional file 1: Table S1a. Associations between SF-36, physical function, pain and background variables. (Univariable linear regression with different subscales of SF-36 as dependent variables). Table S1b. Associations between Qualeffo-41, physical function, pain and background variables. (Univariable linear regression with different subscales of QUALEFFO-41 as dependent variables). [file 12877_2019_1268_MOESM1_ESM.docx]

**Additional file**

**Table a**

Associations between SF-36, physical function, pain and background variables.

(Univariable linear regression with different subscales of SF-36 as dependent variables)

|  | Standardized β | P-value | B (95% CI) |
| --- | --- | --- | --- |
| ***Physical Functioning*** | | | |
| Age | -0.19 | **0.021** | -0.75(-1.14 to -0.12) |
| Education, years | 0.22 | **0.010** | 1.47(0.36 – 2.57) |
| Living condition | 0.05 | 0.518 | 2.52(-5.17 -10.21) |
| Pain | -0.48 | **<0.001** | -4.43(-5.79 to -3.08) |
| BMI | -0.24 | **0.003** | -1.50(-2.49 to -0.51) |
| Comorbidity | 0.23 | **0.005** | 10.66(3.20 – 18.11) |
| Functional reach | 0.46 | **<0.001** | 1.58(1.07 – 2.09) |
| 10 m walking speed | 0.67 | **<0.001** | 51.54(42.15 – 60.93) |
| Arm curl | 0.38 | **<0.001** | 2.25(1.34 – 3.15) |
| 30 sec Sit to Stand | 0.55 | **<0.001** | 3.21(2.40 – 4.02) |
| ***Role Physical*** | | | |
| Age | -0.03 | 0.721 | -0.15(-0.96- 0.66) |
| Education, years | 0.15 | 0.066 | 1.33(-0.09- 2.74) |
| Living condition | 0.02 | 0.853 | 0.91(-8.76 – 10.57) |
| Pain | -0.57 | **<0.001** | -6.69(-8.27 to -5.10) |
| BMI | -0.22 | **0.007** | -1.72(-2.96 to -0.47) |
| Comorbidity | 0.30 | **<0.001** | 17.90(8.71 – 27.09) |
| Functional reach | 0.35 | **<0.001** | 1.54(0.86 – 2.21) |
| 10 m walking speed | 0.54 | **<0.001** | 53.16(37.79 – 66.53) |
| Arm curl | 0.23 | **0.005** | 1.73(0.53 – 2.93) |
| 30 sec Sit to Stand | 0.38 | **<0.001** | 2.84(1.71 – 3.97) |
| ***Bodily Pain*** | | | |
| Age | 0.01 | 0.945 | 0.02(-0.64 – 0.69) |
| Education, years | 0.98 | 0.241 | 0.69(-0.47 – 1.85) |
| Living condition | 0.13 | 0.126 | 6.14(-1.74 – 14.03) |
| Pain | -0.77 | **<0.001** | -7.40(-8.41 to -6.39) |
| BMI | -0.17 | **0.036** | -1.10(-2.13 to – 0.07) |
| Comorbidity | 0.32 | **<0.001** | 15.58(8.12 – 23.03) |
| Functional reach | 0.22 | **0.007** | 0.78(0.21 – 1.35) |
| 10 m walking speed | 0.37 | **<0.001** | 29.33(17.18 – 41.48) |
| Arm curl | 0.18 | **0.026** | 1.13(0.14 – 2.13) |
| 30 sec Sit to Stand | 0.30 | **<0.001** | 1.85(0.90 – 2.98) |
| ***General Health*** | | | |
| Age | 0.08 | 0.330 | 0.32(-0.03 – 0.97) |
| Education, years | 0.08 | 0.354 | 0.53(-0.60 – 1.66) |
| Living condition | -0.01 | 0.920 | -0.40(-8.18 – 7.39) |
| Pain | -0.36 | **<0.001** | -3.34(-4.80 to – 1.88) |
| BMI | 0.00 | 0.967 | -0.02(-1.05 – 101) |
| Comorbidity | 0.24 | **0.004** | 11.27(3.74 – 18.79) |
| Functional reach | 0.17 | **0.039** | 0.60(0.03 – 1.17) |
| 10 m walking speed | 0.34 | **<0.001** | 26.67(14.61 – 38.74) |
| Arm curl | 0.11 | 0.198 | 0.65(-0.34 – 1.63) |
| 30 sec Sit to Stand | 0.26 | **0.001** | 1.56(0.61 – 2.50) |
| ***Vitality*** | | | |
| Age | -0.03 | 0.760 | -0.07(-0.54 – 0.39) |
| Education, years | 0.12 | 0.160 | 0.58(-0.23 – 1.39) |
| Living condition | 0.08 | 0.347 | 2.65(-2.90 – 8.80) |
| Pain | -0.41 | **<0.001** | -2.75(-3.76 to – 1.73) |
| BMI | -0.07 | 0.404 | -0.31(1.04 – 0.42) |
| Comorbidity | 0.33 | **<0.001** | 10.98(5.74- 16.23) |
| Functional reach | 0.24 | **0.004** | 0.59(0.19- 0.99) |
| 10 m walking speed | 0.35 | **<0.001** | 19.36(10.76 – 27.95) |
| Arm curl | 0.15 | 0.073 | 0.65(-0.6 – 1.36) |
| 30 sec Sit to Stand | 0.25 | **0.002** | 1.06(0.38 – 1.73) |
| ***Social Functioning*** | | | |
| Age | -0.06 | 0.463 | -0.21(-0.79 – 0.36) |
| Education, years | 0.16 | 0.055 | 0.98(-0.02 – 1.98) |
| Living condition | 0.20 | **0.018** | 8.04(1.40 – 14.69) |
| Pain | -0.35 | **<0.001** | -2.89(-4.18 to -1.59) |
| BMI | 0.05 | 0.566 | 0.26(-0.64 – 1.17) |
| Comorbidity | 0.32 | **<0.001** | 13.31(6.83 – 19.78) |
| Functional reach | 0.14 | 0.104 | 0.42(-0.87 – 0.92) |
| 10 m walking speed | 0.33 | **<0.001** | 22.94(12.27 – 33.60) |
| Arm curl | 0.14 | 0.080 | 0.77(-0.10 – 1.64) |
| 30 sec Sit to Stand | 0.19 | **0.021** | 1.00(0.15 – 1.85) |
| ***Role Emotional*** | | | |
| Age | -017 | 0.041 | -0.59(-1.16 to -0.02) |
| Education, years | 0.22 | **0.008** | 1.43(0.35 – 2.32) |
| Living condition | 0.18 | **0.030** | 7.44(0.71 – 14.17) |
| Pain | -0.32 | **<0.001** | -2.70(-4.01 to -1.39) |
| BMI | -0.12 | 0.145 | 0.67(-1.57 – 0.23) |
| Comorbidity | 0.23 | **0.005** | 9.56(2.90 – 16.23) |
| Functional reach | 0.14 | 0.102 | 0.42(-0.08 – 0.92) |
| 10 m walking speed | 0.42 | **<0.001** | 28.86(18.56 – 39.15) |
| Arm curl | 0.16 | **0.045** | 0.88(0.09 – 1.75) |
| 30 sec Sit to Stand | 0.21 | **0.010** | 1.11(0.27 – 1.96) |
| ***Mental Health*** | | | |
| Age | -0.15 | 0.070 | -0.23(-1.15 – 0.05) |
| Education, years | 0.08 | 0.355 | 0.64(-0.57 – 1.57) |
| Living condition | 0.18 | **0.036** | 4.67(0.32 – 9.02) |
| Pain | -0.24 | **0.004** | -1.27(-2.13 to -0.41) |
| BMI | 0.01 | 0.923 | 0.13(-0.91 – 1.01) |
| Comorbidity | 0.21 | **0.012** | 6.81(2.06 – 16.16) |
| Functional reach | 0.02 | 0.855 | 0.08(-0.49 – 0.59) |
| 10 m walking speed | 0.22 | **0.006** | 10.83(4.61 – 27.82) |
| Arm curl | 0.19 | **0.021** | 0.73(0.17 – 2.00) |
| 30 sec Sit to Stand | 0.13 | 0.129 | 0.40(-0.21 – 1.60) |
| ***Physical Component Score*** | | | |
| Age | -0.03 | 0.723 | -0.05(-0.33-0.23) |
| Education, years | 0.02 | 0.842 | 0.34(-3.03-3.70) |
| Living condition | 0.14 | 0.102 | 0.41(-0.08-0.90) |
| Pain | 0.66 | **<0.001** | -2.66(-3.16 to-2.15) |
| BMI | -0.22 | **0.007** | -0.60(-1.04 to -0.16) |
| Comorbidity | 0.30 | **<0.001** | 6.09(2.90-9.28) |
| Functional reach | 0.42 | **<0.001** | 0.66(0.42 – 0.89) |
| 10 m walking speed | 0.58 | **<0.001** | 19.47(14.97- 23.97) |
| Arm curl | 0.26 | **<0.001** | 0.69(0.28 – 1.11) |
| 30 sec Sit to Stand | 0.48 | **<0.001** | 1.23(0.86 – 1.60) |
| ***Mental Component Score*** | | | |
| Age | -0.11 | 0.193 | -0.12(-0.31 – 0.06) |
| Education, years | 0.13 | 0.113 | 0.26(-0.06 – 0.58) |
| Living condition | 0.22 | **0.008** | 2.97(0.79 – 5.15) |
| Pain | -0.03 | 0.719 | -0.08(-0.53 – 0.37) |
| BMI | 0.12 | 0.140 | 0.22(-0.07-0.51) |
| Comorbidity | 0.20 | **0.016** | 2.67(0.51- 4.83) |
| Functional reach | -0.09 | 0.262 | -0.10(-0.27 – 0.07) |
| 10 m walking speed | 0.05 | 0.533 | 1.15(-2.48 – 4.78) |
| Arm curl | 0.04 | 0.630 | 0.07(-0.22 – 0.35) |
| 30 sec Sit to Stand | -0.08 | 0.345 | -0.13(-0.41 – 0.14) |

**Table b**

Associations between Qualeffo-41, physical function, pain and background variables.

(Univariable linear regression with different subscales of QUALEFFO-41 as dependent variables.)

|  | Standardized β | P-value | B (95% CI) |
| --- | --- | --- | --- |
| ***Score Pain*** | | | |
| Age | -0.08 | 0.323 | -0.35(-1.06 – 0.35) |
| Education, years | -0.05 | 0.546 | -0.38(-1.62 – 0.86) |
| Living condition | -0.03 | 0.768 | -1.25(-9.63 – 7.12) |
| Pain | 0.76 | **<0.001** | 7.70(6.60-8.81) |
| BMI | 0.23 | **0.005** | 1.59(0.49 – 2.69) |
| Comorbidity | -0.36 | **<0.001** | -18.55(-26.36to-0.76) |
| Functional reach | -0.16 | 0.051 | 0.61(-1.22 – 0.00) |
| 10 m walking speed | -0.27 | **0.001** | -22.95(-36.27to-9.62) |
| Arm curl | -0.16 | 0.052 | -1.05(-2.11 – 0.01) |
| 30 sec Sit to Stand | -0.21 | **0.011** | -1.35(-2.39 –0.32) |
| ***Physical function*** | | | |
| Age | 0.21 | **0.009** | 0.48(0.12 – 0.85) |
| Education, years | -0.24 | **0.004** | -0.94(-1.57 to -0.31) |
| Living condition | -0.13 | 0.110 | -3.55(-7.91 – 0.81) |
| Pain | 0.55 | **<0.001** | 2.88(2.15 – 3.61) |
| BMI | 0.28 | **0.001** | 0.99(0.43 – 1.55) |
| Comorbidity | -0.30 | **<0.001** | -7.98(-12.19 to -3.78) |
| Functional reach | -0.45 | **<0.001** | 0.90(-1.19 to – 0.61) |
| 10 m walking speed | -0.62 | **<0.001** | -27.88(-33.56 to-22.19) |
| Arm curl | -0.30 | **<0.001** | -1.04(-1.58 to -0.50) |
| 30 sec Sit to Stand | -0.50 | **<0.001** | -1.70(-2.18 to -1.22) |
| ***Score Leisure and Social Activities*** | | | |
| Age | 0.12 | 0.150 | 0.44(-0.16 – 1.04) |
| Education, years | -0.32 | **0.000** | -2.02(-3.02 to -1.02) |
| Living condition | -0.04 | 0.660 | -1.61(-8.82 -5.60) |
| Pain | 0.46 | **<0.001** | 3.96(2.66 – 5.26) |
| BMI | 0.11 | 0.210 | 0.60(-0.34 – 1.55) |
| Comorbidity | -0.14 | 0.101 | -5.92(-13.01 – 1.17) |
| Functional reach | -0.45 | **<0.001** | -1.45(-1.94 to -0.97) |
| 10 m walking speed | -0.61 | **<0.001** | -44.26(-53.87 to-34.65) |
| Arm curl | -0.36 | **<0.001** | -1.96(-2.80 to – 1.11) |
| 30 sec Sit to Stand | -0.51 | **<0.001** | -2.86(-3.67 to -2.06) |
| ***Score Views about Life in General*** | | | |
| Age | 0.00 | 0.983 | -0.01(-0.67 – 0.66) |
| Education, years | -0.19 | **0.031** | -1.23(-2.35 to -0.11) |
| Living condition | -0.15 | 0.092 | -6.61(-14.31 -1.10) |
| Pain | 0.43 | **<0.001** | 3.92(2.52 -5.31) |
| BMI | 0.15 | 0.091 | 0.87(-0.14 -1.88) |
| Comorbidity | -0.28 | **0.001** | -12.86(-20.42to-5.23) |
| Functional reach | -0.21 | **0.016** | -0.71(-1.28 to -0.13) |
| 10 m walking speed | -0.41 | **<0.001** | -31.38(-42.86 to-19.90) |
| Arm curl | -0.12 | 0.146 | -0.73(-1.72 -0.26) |
| 30 sec Sit to Stand | -0.27 | **0.002** | -1.50(-2.43 to -0.58) |
| ***Score Mood*** | | | |
| Age | 0.07 | 0.410 | 0.15(-0.21-0.51) |
| Education, years | -0.13 | 0.133 | -0.48(-1.12-0.1)5 |
| Living condition | -0.20 | **0.015** | -5.29(-9.55 to – 1.03) |
| Pain | 0.28 | **0.001** | 1.43(0.60 – 2.26) |
| BMI | -0.11 | 0.197 | -0.37(-0.94 – 0.20) |
| Comorbidity | -0.16 | 0.057 | -4.10(-8.34 -0.13) |
| Functional reach | -0.16 | 0.056 | -0.32(-0.65 to -0.01) |
| 10 m walking speed | -0.24 | **0.004** | -10.29(-17.20 to -3.37) |
| Arm curl | -0.11 | 0.165 | -0.39(-0.93 – 0.16) |
| 30 sec Sit to Stand | -0.15 | 0.076 | -0.49(-1.03 – 0.05) |
| ***Total Score*** | | | |
| Age | 0.12 | 0.141 | 0.27(-0.09 -0.64) |
| Education, years | -0.25 | **0.002** | -0.98(-1.60 to -0.35) |
| Living condition | -0.20 | **0.015** | -3.85(-9.55 to -1.03) |
| Pain | 0.65 | **<0.001** | 3.40(2.73 – 4.07) |
| BMI | 0.20 | **0.018** | 0.69(0.21 – 1.26) |
| Comorbidity | -0.32 | **<0.001** | -8.51(-12.66 to -4.37) |
| Functional reach | -0.41 | **<0.001** | -0.82(-1.11 to -0.52) |
| 10 m walking speed | -0.58 | **<0.001** | -26.27)(-32.09 to-20.46) |
| Arm curl | -0.30 | **<0.001** | -1.03(-1.56 to -0.50) |
| 30 sec Sit to Stand | -0.47 | **<0.001** | -1.57(-2.06 to -1.08) |
